# Distinct Expression Patterns of Osteopontin and Dentin Matrix Protein 1 Genes in Pituitary Gonadotrophs

**DOI:** 10.3389/fendo.2019.00248

**Published:** 2019-04-17

**Authors:** Ivana Bjelobaba, Marija M. Janjic, Rafael Maso Prévide, Daniel Abebe, Marek Kucka, Stanko S. Stojilkovic

**Affiliations:** ^1^Eunice Kennedy Shriver National Institute of Child Health and Human Development, National Institutes of Health (NIH), Bethesda, MD, United States; ^2^Institute for Biological Research Sinisa Stankovic, University of Belgrade, Belgrade, Serbia

**Keywords:** pituitary, gonadotrophs, SIBLINGs, *Spp1*, osteopontin, *Dmp1*, GnRH, cell-matrix

## Abstract

Cell-matrix interactions play important roles in pituitary development, physiology, and pathogenesis. In other tissues, a family of non-collagenous proteins, termed SIBLINGs, are known to contribute to cell-matrix interactions. Anterior pituitary gland expresses two SIBLING genes, *Dmp1* (dentin matrix protein-1) and *Spp1* (secreted phosphoprotein-1) encoding DMP1 and osteopontin proteins, respectively, but their expression pattern and roles in pituitary functions have not been clarified. Here we provide novel evidence supporting the conclusion that *Spp1/*osteopontin, like *Dmp1*/DMP1, are expressed in gonadotrophs in a sex- and age-specific manner. Other anterior pituitary cell types do not express these genes. In contrast to *Dmp1, Spp1* expression is higher in males; in females, the expression reaches the peak during the diestrus phase of estrous cycle. In further contrast to *Dmp1* and marker genes for gonadotrophs, the expression of *Spp1* is not regulated by gonadotropin-releasing hormone *in vivo* and *in vitro*. However, *Spp1* expression increases progressively after pituitary cell dispersion in both female and male cultures. We may speculate that gonadotrophs signal to other pituitary cell types about changes in the structure of pituitary cell-matrix network by osteopontin, a function consistent with the role of this secretory protein in postnatal tissue remodeling, extracellular matrix reorganization after injury, and tumorigenesis.

## Introduction

Cell—extracellular matrix (ECM) tridimensional network is critical for the proper functioning of all tissues (1), including anterior pituitary gland (2). Individual components of ECM include two main classes of macromolecules; proteoglycans and fibrous proteins (laminin, collagens, elastins, and fibronectin) (3, 4). The effects of the ECM are mediated mainly by plasma membrane receptors called integrins; individual components of ECM bind to different integrins, leading to activation of multiple signaling pathways (5). In anterior pituitary, the presence of ECM molecules, like collagens, laminin, and small leucine-rich proteoglycans, and cell types producing these proteins have been identified (6–10). Pituitary cells also express integrins (11). There is increasing evidence that ECM is critical for development and differentiation of the pituitary gland (12), for postnatal cell migration and pituitary remodeling (13), and for hormone secretion (14, 15). As in other tissues, ECM molecules may also have important roles in pituitary tumorigenesis (16).

In addition to proteoglycans and fibrous proteins, ECM contains other proteins, including SIBLINGs (Small Integrin-Binding Ligand, N-linked Glycophosphoproteins). SIBLINGs are encoded by a family of five genes, comprising secreted phosphoprotein 1 (*Spp1*), which encodes osteopontin (OPN), integrin-binding sialoprotein, which encodes bone sialoprotein, and dentin matrix protein 1 (*Dmp1*), dentin sialophosphoprotein and matrix extracellular phosphoglycoprotein, which encode proteins with the same name (17). SIBLINGs are soluble, secreted proteins that can act as modulators of cell adhesion as well as autocrine and paracrine ligands for ECM receptors. For example, OPN activates a variety of integrin receptors as well as CD44 receptor splice variants (18). The ligand activities of SIBLINGs are modulated by post-translational modifications, such as phosphorylation, glycosylation, proteolytic processing, sulphation, and transglutaminase cross-linking (19, 20).

SIBLINGs were initially described as mineralized tissue-associated genes (21). However, recent findings indicate that they are more widely distributed, including normal ductal epithelia in salivary gland (22) and kidney (23). *Spp1*/OPN were also detected in central nervous system (24), where they may play a role in neurodegenerative diseases, such as Alzheimer's disease (25), Parkinson's disease (26), and multiple sclerosis (27, 28). *Dmp1*/DMP1 was reported to be expressed in the brain, as well as in the liver, muscle, pancreas and kidney (29). SIBLING gene family is also expressed in various tumors (18, 30) and OPN was suggested to be a valuable biomarker for diagnosing and treating cancers (31).

Our recent RNA-sequence analysis revealed that *Dmp1* and *Spp1*, but no other SIBLING genes, were also expressed in anterior pituitary cells (32). The expression of *Dmp1* is restricted to gonadotrophs, cells that produce luteinizing hormone (LH) and follicle-stimulating hormone (FSH), and is stimulated by gonadotropin-releasing hormone (GnRH) but not by other hypothalamic releasing factors. GnRH-induced expression of this gene is coupled with release of DMP1 in extracellular medium through the regulated secretory pathway. *In vivo*, the sex-specific pituitary *Dmp1* expression is established during the peripubertal period and is elevated after ovulation. GnRH induction of *Dmp1* is mediated by the protein kinase C signaling pathway through ERK1/2 signaling pathway; in addition, the response is facilitated by progesterone (32). It has also been shown that *Spp1* is expressed in gonadotrophs and that mRNA levels were down regulated in anterior pituitary of lactating animals and by injection of estradiol (33).

Here we summarize work on *Spp1* expression in rat anterior pituitary cells *in vivo* and *in vitro*. These include sexual dimorphism in *Spp1* expression during maturation, effects of cell-matrix network destruction by cell dispersion procedure on *Spp1* expression, and evaluation of the role of GnRH receptors (GnRHR) in the expression of this gene. We also studied the expression pattern of OPN in prepubertal females and males and cycling females as well as the cell type specificity in expression of this protein. Finally, we compared *Spp1* expression with *Dmp1* expression in pituitary gonadotrophs.

## Methods

### Animals

Experiments were performed with female and male Sprague Dawley rats obtained from Taconic Farms (Germantown, NY). Animals were housed under constant conditions of temperature and humidity, with light on between 6 a.m. and 8 p.m. All experiments were repeated at least three times and were approved by the NICHD Animal Care and Use Committee (16-041).

### Ontogeny of *Spp1*/OPN Expression

Experiments were performed with 2 days to 12 weeks old female and male rats. In some postpubertal females, a vaginal smear was taken of adult females to obtain information about the estrous cycle stage. Vaginal material was stained by a 0.1% aqueous solution of methylene blue and examined under a microscope. Animals were euthanized via asphyxiation with CO_2_ and whole pituitary or anterior pituitary glands were removed and used for histological preparations or RNA extraction as described below.

### Anterior Pituitary Cell Culture

For *in vitro* experiments, 4- or 7-week-old female and male rats were euthanized in the morning. After decapitation anterior pituitary glands were removed and pituitary cells were mechanically dispersed after trypsin and EDTA treatments as previously described (34). Dispersed cells were seeded on poly-D-lysine coated 24-well plates, 1.5 million per well. Plated cells were initially cultured in medium 199 containing Earle's salts, sodium bicarbonate, penicillin (100 units per ml), streptomycin (100 μg per ml) and 10% heat-inactivated horse serum (Life Technologies, Grand Island, NY). If not otherwise specified, experiments were performed with cells cultured overnight, washed and bathed in medium 199 with Hank's salt and containing 0.1% BSA. At the end of experiments, attached cells were scraped for RNA extraction.

### *In Vivo* Treatments

Four- or seven-week-old female and male rats were injected once intraperitoneally with a GnRHR agonist, buserelin acetate (5 μg/0.4 ml/per animal) from Sigma (St. Louis, MO) or PBS (0.4 ml/per animal). Euthanasia was performed 3, 6, or 9 h after intraperitoneal injections. After decapitation, blood was collected, and serum was separated and stored at −80°C for LH concentration measurement. The whole anterior pituitaries were collected in RNA later stabilization solution (Thermo Fisher Scientific, Waltham, MA) for RNA extraction.

### qRT-PCR Analysis

Total RNA was extracted from individual anterior pituitary glands and primary cultures of anterior pituitary cells using RNeasy Plus Mini Kit (Qiagen, Valencia, CA). RNA was reverse transcribed with a Transcriptor First Stand cDNA Synthesis Kit (Roche Applied Sciences, Indianapolis, IN). Quantitative RT-PCR was performed using Applied Biosystems pre-designed Taq-Man Gene Expression Assays for rats using the LightCycler® TaqMan® Master Mix and the LightCycler 2.0 Real-time PCR system (Roche Applied Science). Target gene expression levels were determined by the comparative 2^∧^(-delta C(T)) quantification method using *Gapdh* as the reference gene, which was previously established to be a suitable reference gene for the anterior pituitary tissue (35). Applied Biosystems predesigned TaqMan Gene Expression Assays were used: *Dmp1*: Rn01450122_m1, *Spp1* (Rn00681031_m1), *Gnrhr* (Rn00578981_m1), and *Gapdh*: Rn01462662_g1.

### Immunohistochemical Analysis

Whole pituitaries were quickly and carefully isolated and fixed in Bouin's solution for 48 h. Tissue was then embedded in paraffin and cut in coronal plane. Five μm thick sections were mounted on glass slides and processed for immunohistochemistry as previously described (36, 37). Briefly, after deparaffinization, antigen retrieval in citrate buffer (0.01 M, pH 6) was performed. Monoclonal OPN antibody (The Developmental Studies Hybridoma Bank, Iowa City, IA) in 1:400 dilution was applied overnight at 4°C. Secondary donkey anti-mouse-HRP (Santa Cruz Biotechnology, Dallas, TX) was then applied at 1:200 dilution for 2 h, and visualization was afterwards performed with diaminobenzidine tetrahydrochloride (Vector Laboratories, Burlingame, CA). Slides were mounted with DPX (Sigma, St. Louis, MO) and sections examined under an Olympus BX61 microscope. For double immunofluorescence studies, after incubation of sections with the OPN antibody, secondary Alexa Fluor donkey-anti-mouse 488 (Thermo Fisher Scientific, Waltham, MA) was applied at 1:400 dilution for 2 h. Sections were then incubated for 2 h with rabbit-anti rat LH or guinea pig-anti FSH (1:500 dilution) obtained from Dr. A. F. Parlow (National Institute of Diabetes and Digestive and Kidney Diseases, National Hormone and Peptide Program, Torrance, CA). Following the incubation with donkey-anti rabbit or donkey-anti guinea pig 555 Alexa Fluor secondary antibodies (1:400 dilution) slides were mounted with Mowiol based mounting medium and examined under inverted Zeiss LSM 510 confocal microscope. Triple immunofluorescence labeling was done as described above using Alexa Fluor Dyes: donkey-anti rabbit 488, donkey-anti guinea pig 555, and donkey-anti mouse 647 (Thermo Fisher Scientific, Waltham, MA), and the sections were examined under Leica TCS SP5 II confocal microscope.

### Statistics

All numerical values in the text are reported as the mean ± SEM from one of at least three similar *in vivo* or *in vitro* experiments. KaleidaGraph Program (Synergy Software, Reading, Pennsylvania) was used for all calculation and graph presentation. Significant differences between means were determined by a Student's *t*-test or an ANOVA accompanied with the *post hoc* Student-Newman-Keuls test as well as for regression/correlation analyses and calculation of the half time of decay in gene expression. *P*-values of <0.05 were considered significant.

## Results

### *Spp1* Is Expressed in Anterior Pituitary of Developing Animals in a Sex-Specific Manner

**Figure 1:**
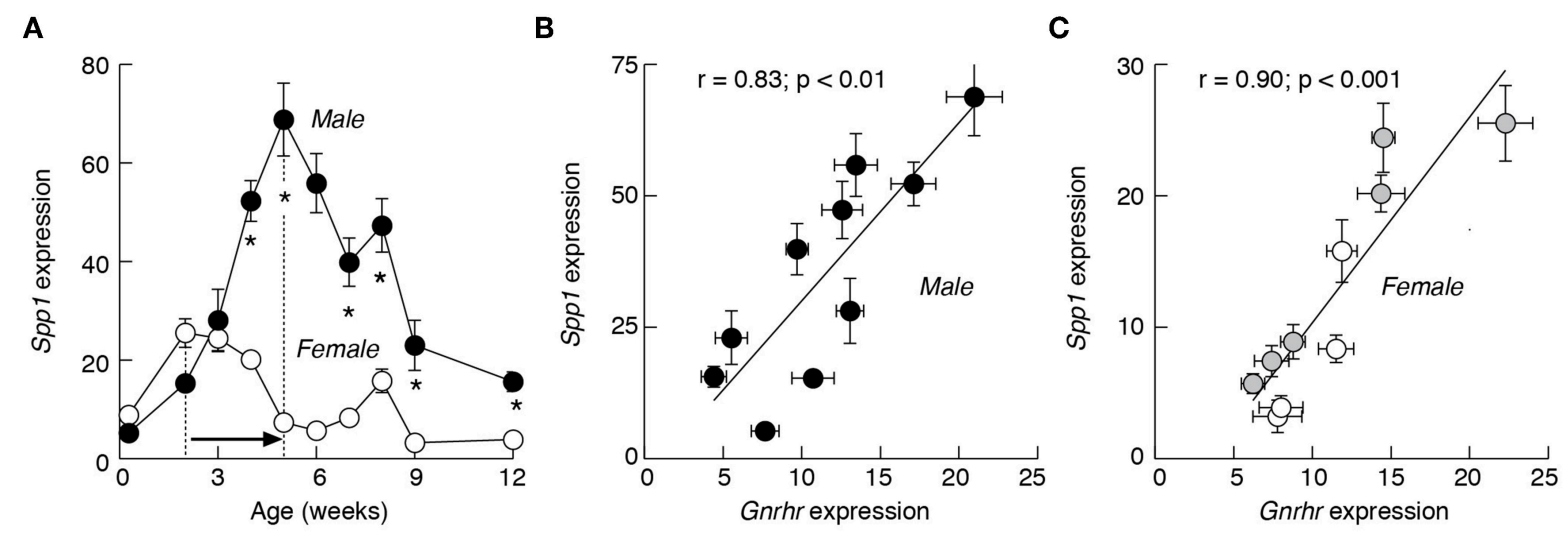
The expression pattern of *Spp1* mRNA in anterior pituitary of developing rats is comparable to *Gnrhr* expression. **(A)** The sex-specific developmental profiles of *Spp1* expression. White circles: females; black circles: males. In postpubertal females, the mean values are derived from regularly cycling animals in proestrus, estrus, and diestrus 1 and 2 stages of the cycle. **(B,C)** The expression of *Spp1* correlates with expression of *Gnrhr*, a gonadotroph marker gene, in male **(B)** and female **(C)** pituitaries. Data points shown are mean ± SEM values from 6 to 37 animals per group, relative to *Gapdh* (set as 100%). Correlation and linear regression analyses and statistical evaluation are described in Material and Methods; *r*, coefficient of correlation. The mean ± SEM values for *Gnrhr* are derived from (38). Asterisks indicate significant differences between pairs **(A)**; the *p* values for *r* coefficient are shown on top of panels **(B,C)**. Gray circles, prepubertal females; white circle, postpubertal females **(C)**.

The *Spp1* expression was investigated in male and female anterior pituitary tissue from 2 days to 12 weeks old rats (Figure 1A). During this period, the gene was expressed in both sexes and the mRNA expression varied between 5 and 70% of the expression of *Gapdh*, a housekeeping gene. Pituitaries obtained from animals up to 3 weeks of age showed no sex difference in the expression of *Spp1*. From week four onward, however, the sex-specific expression pattern was established. First, *Spp1* levels were always significantly higher in male pituitaries. Second, there were differences in terms of timing needed to reach the peak in mRNA expression. In males, there was a progressive increase in *Spp1* expression, reaching the peak value at 5 weeks of age, with ~13-fold increase in expression when compared to the second day of age. This was followed by a progressive decrease in gene expression during peripubertal and postpubertal periods. In females, however, the first peak in expression was reached during infantile period, with an ~3-fold increase compared to the second day of age. This was followed by a gradual decline during the juvenile, peripubertal and postpubertal age, with a secondary transient increase in expression at the age of 8 weeks (Figure 1A).

In both female and male rats, the developmental profiles of *Spp1* were highly comparable to profiles of major gonadotroph-specific (hereafter marker) genes, *Gnrhr, Lhb*, and *Fshb*, as well as to the gonadotroph/thyrotroph-specific gene *Cga* (35). This prompted us to examine the relationship between *Spp1* expression vs. marker gene expression during sexual maturation. This was done using a linear correlation analysis and the Pearson *r*-coefficient as an indicator of significance of correlation. Scatter data points for *Gnrhr* vs. *Spp1* in developing males (Figure 1B) and females (Figure 1C), had a linear tendency, with the *r* value significant in both cases.

**Figure 2:**
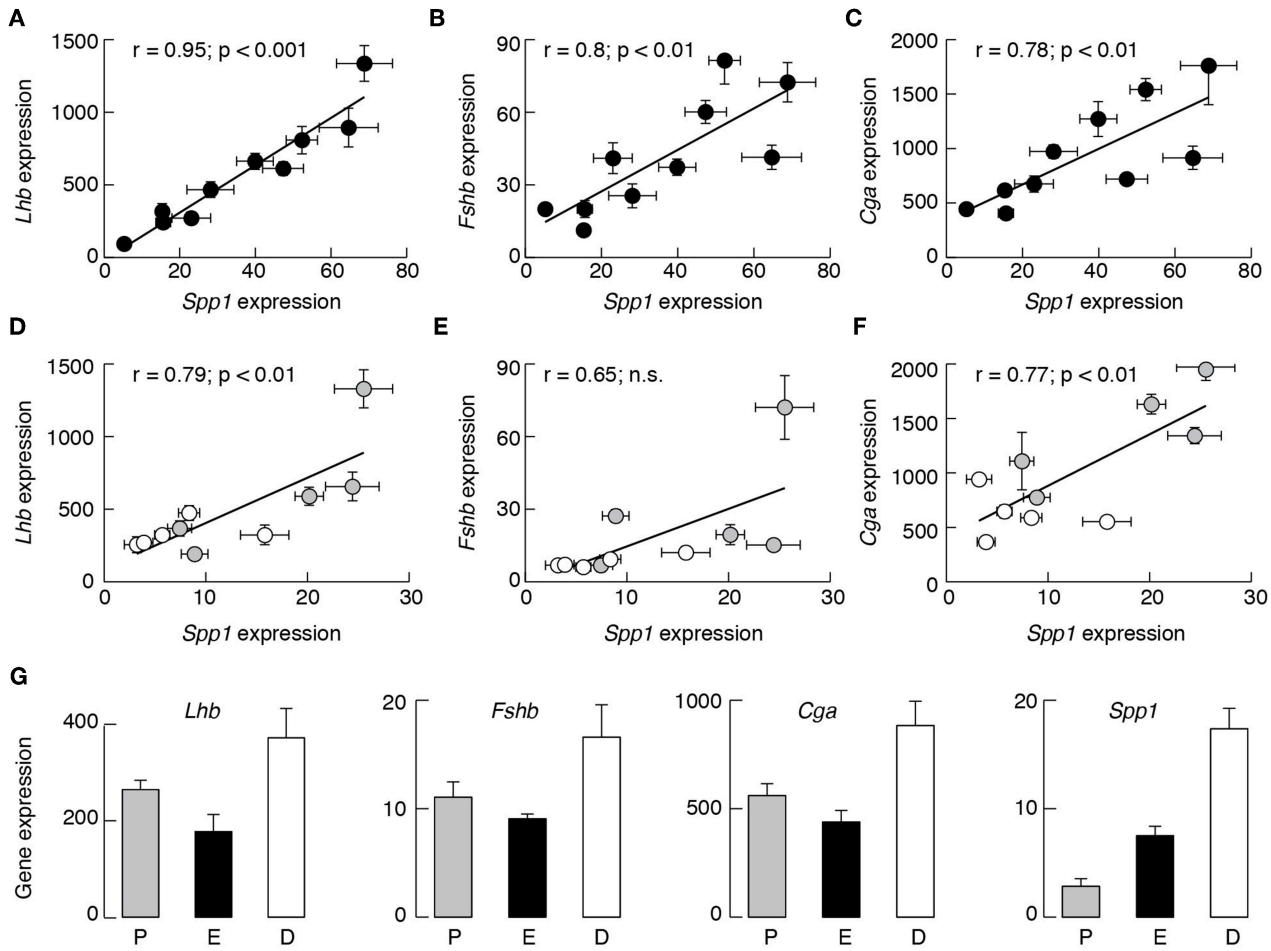
The expression of *Spp1* correlates with expression of other gonadotroph marker genes in a sex-specific manner in anterior pituitary tissue from developing rats. Correlation between *Spp1* vs. *Lhb* **(A,D)**, *Fshb* **(B,E)**, and *Cga* **(C,F)** expression. Data points for *Lhb, Fshb*, and *Cga* are derived from (35). n.s., non-significant. Gray circles, prepubertal females; white circles, postpubertal females. **(G)** The expression of gonadotroph marker genes and *Spp1* in postpubertal females during estrous cycle: P, proestrus; E, estrus; D, diestrus-1 (metestrus) + diestrus-2.

Furthermore, *Spp1* expression correlated well with the expression of *Lhb, Fshb*, and *Cga* in males (Figures 2A–C). There was also correlation between *Spp1* vs. *Cga* in females (Figure 2F), with comparable *r*-coefficient values in females and males, as well as between *Spp1* and *Lhb* expression (Figure 2D), but with lower *r*-coefficient in females when compared with males. Finally, the *r*-coefficient value was not significant when *Spp1* expression was compared with *Fshb* expression in females (Figure 2E). The data points from prepubertal females (shown in gray) and in postpubertal females (shown in white) suggest that correlation was attenuated in postpubertal animals.

To clarify how the estrous cycle influences gene expression, we examined *Lhb, Fshb, Cga*, and *Spp1* expression in pituitaries from proestrus, estrus, and combined diestrus-1 (metestrus) and diestrus-2 animals. The expression of *Lhb, Fshb*, and *Cga* was highest in diestrus animals and lowest in estrus animals (Figure 2G). The pattern of *Spp1* expression during of estrous cycle was different: the smallest was during proestrus (2.84 ± 0.70, *n* = 14), followed by estrus (7.50 ± 0.89, *n* = 8; *P* < 0.01 vs. proestrus) and the largest was during the diestrus (17.36 ± 1.89, *n* = 15; *P* < 0.01 vs. proestrus).

In contrast to gonadotroph marker genes, no correlation was observed between *Spp1* expression vs. expression of *Pomc*, a marker gene for corticotrophs and melanotrophs, *Tshb*, a marker gene for thyrotrophs, *Gh1*, a marker gene for somatotrophs, and *Prl*, a marker gene for lactotrophs (data not shown).

These results suggest that *Spp1* is expressed in pituitary gland in a sex-specific manner and that expression of this gene during development is synchronized with expression of gonadotroph signature genes, a finding consistent with a hypothesis that this gene is active only in gonadotrophs. However, the expression of *Spp1* is regulated differently than the expression of gonadotroph marker genes during the estrous cycle.

### *In vivo* OPN Is Specifically Expressed in Pituitary Gonadotrophs

**Figure 3:**
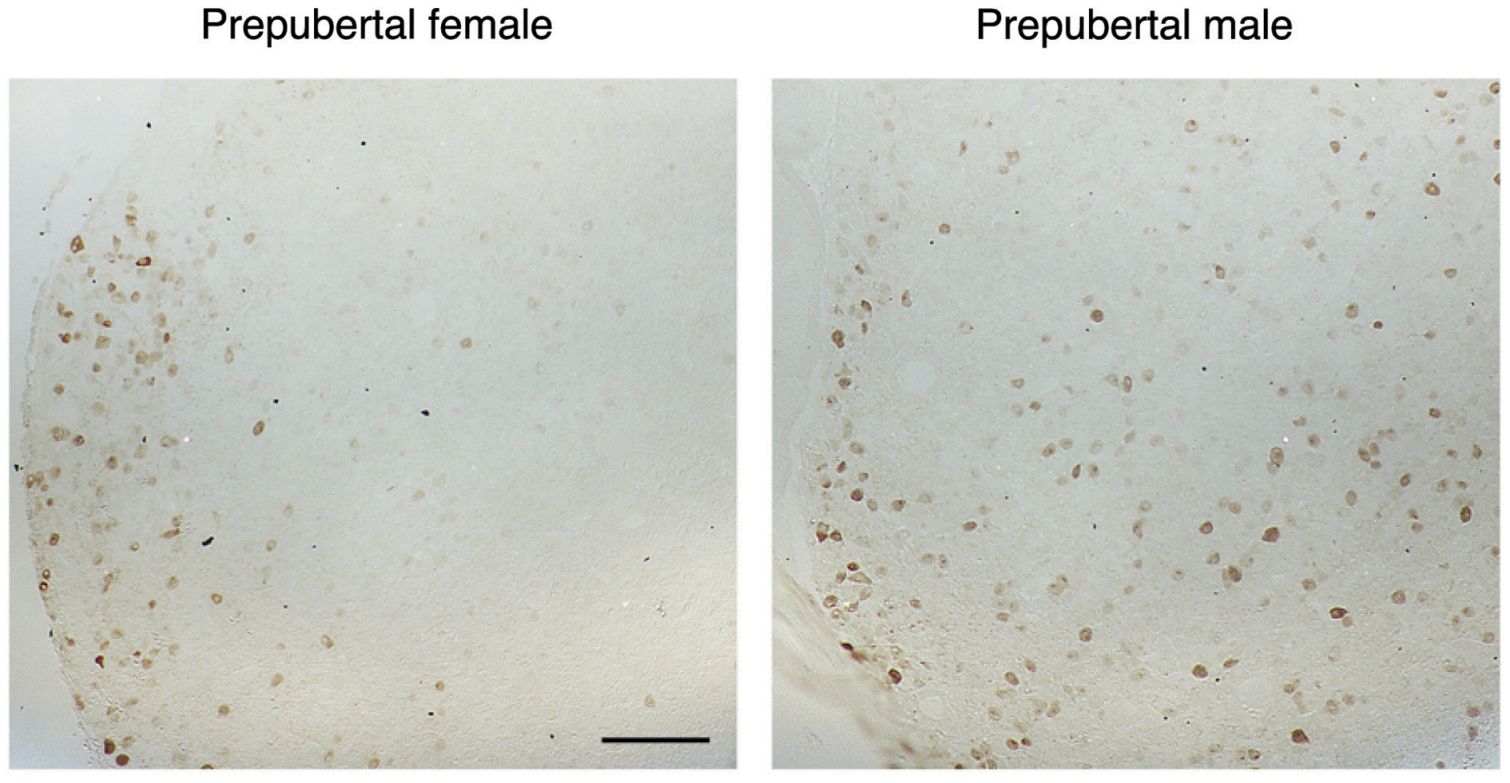
The sex-specific expression pattern of osteopontin (OPN) in the anterior pituitary tissue from prepubertal female and male rats. Male tissue sections contained greater number of labeled cells which were more homogenously distributed, when compared to female pituitary tissue. Scale bar of 200 μm applies to both images.

**Figure 4:**
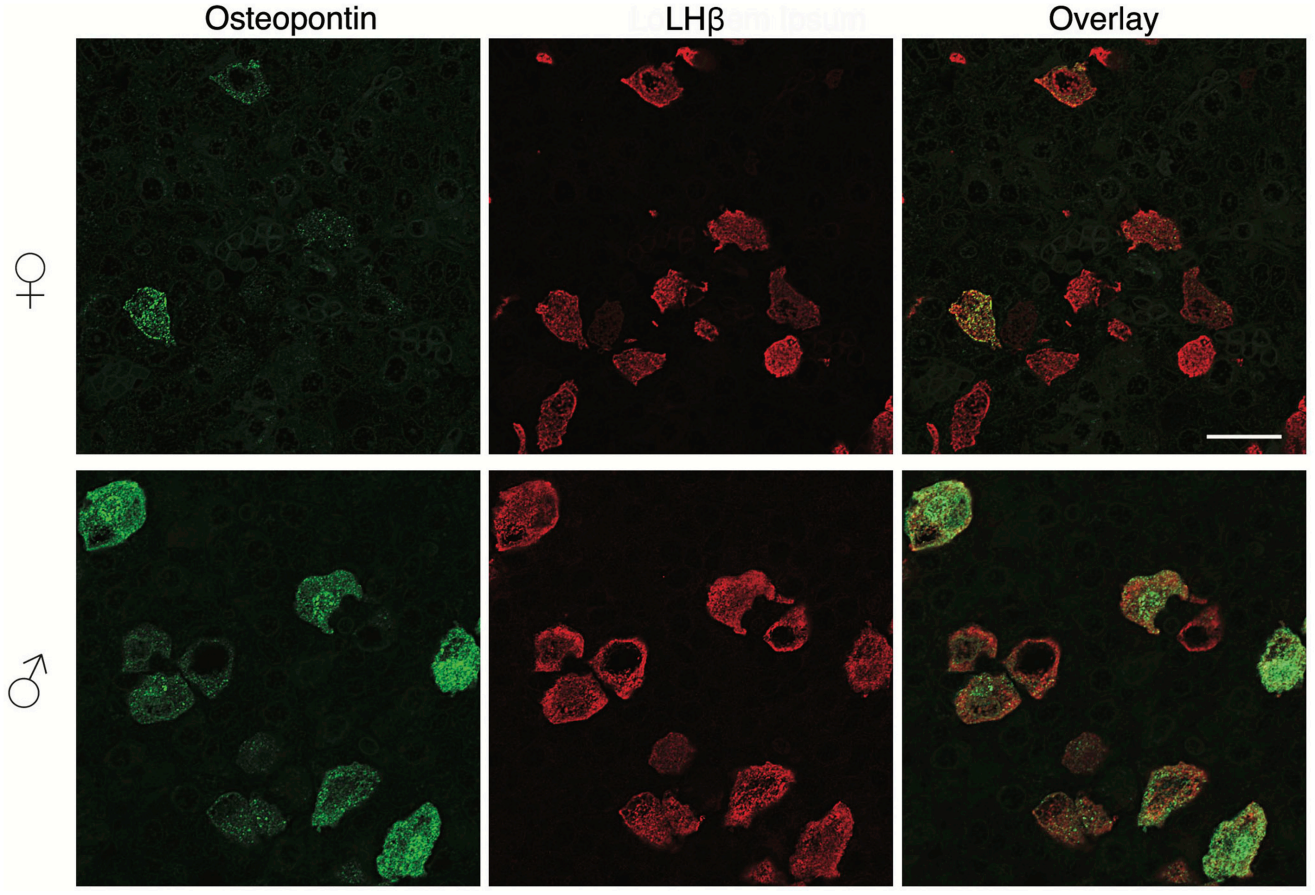
OPN expression in the adult pituitary tissue is sex-specific and restricted to gonadotrophs. Immunofluorescence staining for OPN and LHβ in the anterior pituitary tissue from adult female (top) and male (bottom) rats. Scale bar of 20 μm applies to all images.

**Figure 5:**
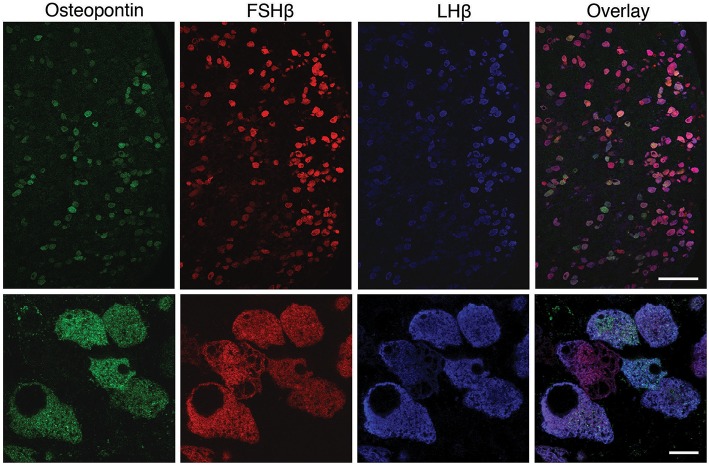
Triple immunofluorescence labeling for OPN (green), FSHβ (red), and LHβ (blue) in adult male rat pituitary tissue. Low magnification images (upper row) show that all OPN-positive cells were also LHβ-positive; most of the OPN-positive cells are both FSHβ and LHβ-positive. However, OPN was not visible in all gonadotrophs. Scale bar: 100 μm. High -magnification of triple immunofluorescence labeling for OPN, FSHβ and LHβ (bottom row) shows a cluster of gonadotroph cells, most of which show immunoreactivity for all three markers. Scale bar: 10 μm.

To clarify this hypothesis, we performed an immunohistochemical analysis of pituitary tissue using antibodies specific for OPN, a protein encoded by *Spp1*, LHβ, and FSHβ. This analysis confirmed that OPN was also present in both female and male pituitary cells during sexual maturation. Figure 3 shows OPN-positive cells in female and male pituitaries from prepubertal animals, with more positive cells observed in male pituitaries. In parallel to mRNA expression, OPN-positive cells were also visible in peripubertal and postpubertal anterior pituitaries from both sexes and with greater number of labeled cells in male tissue sections (data not shown). Finally, double immunohistochemical labeling indicated that all OPN-positive cells in males and females were also LHβ positive, i.e., that *Spp1*/OPN are specifically expressed in LHβ-positive gonadotrophs (Figure 4). Finally, triple immunohistochemical labeling indicated that most of the OPN positive cells in males were positive for both LHβ and FSHβ (Figure 5).

**Figure 6:**
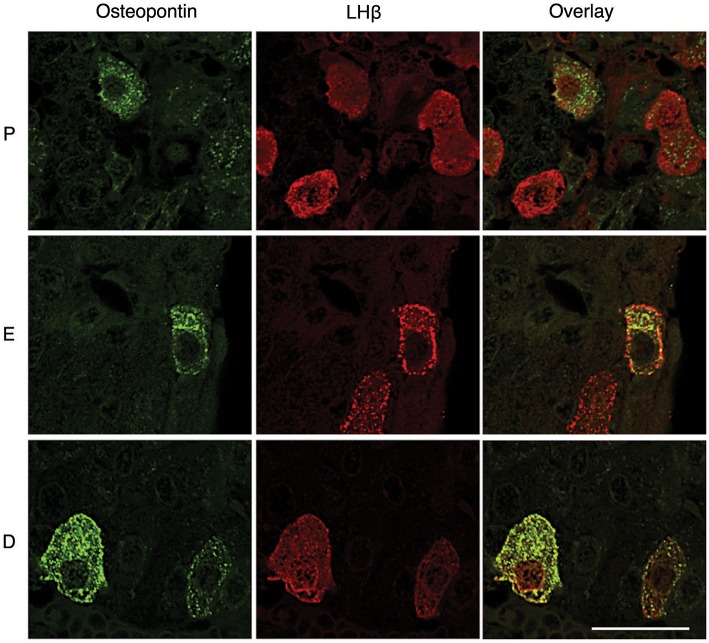
Estrous cycle influences OPN expression in the pituitary tissue. Gonadotrophs were more intensively labeled in diestrus (D), when compared to proestrus (P) and estrus (E). Scale bar = 20 μm applies to all images.

However, OPN labeling was not visible in all gonadotrophs (Figures 4, 5), suggesting that *in vivo* expression of OPN was bellow detection by immunohistochemistry in a fraction of these cells. In parallel to *Spp1* expression during estrous cycle (Figure 2G), greater number of OPN-positive gonadotrophs could be observed in diestrus when compared to other stages of the estrous cycle. Furthermore, OPN-positive gonadotrophs were more intensely labeled in diestrus (Figure 6). Finally, the difference in the expression of gonadotroph marker genes and *Spp1* during estrous cycle suggest that *Spp1* expression was probably independent of the status of GnRH secretion.

### Basal *Spp1* Expression Is Upregulated in Cultured Pituitary Cells

**Figure 7:**
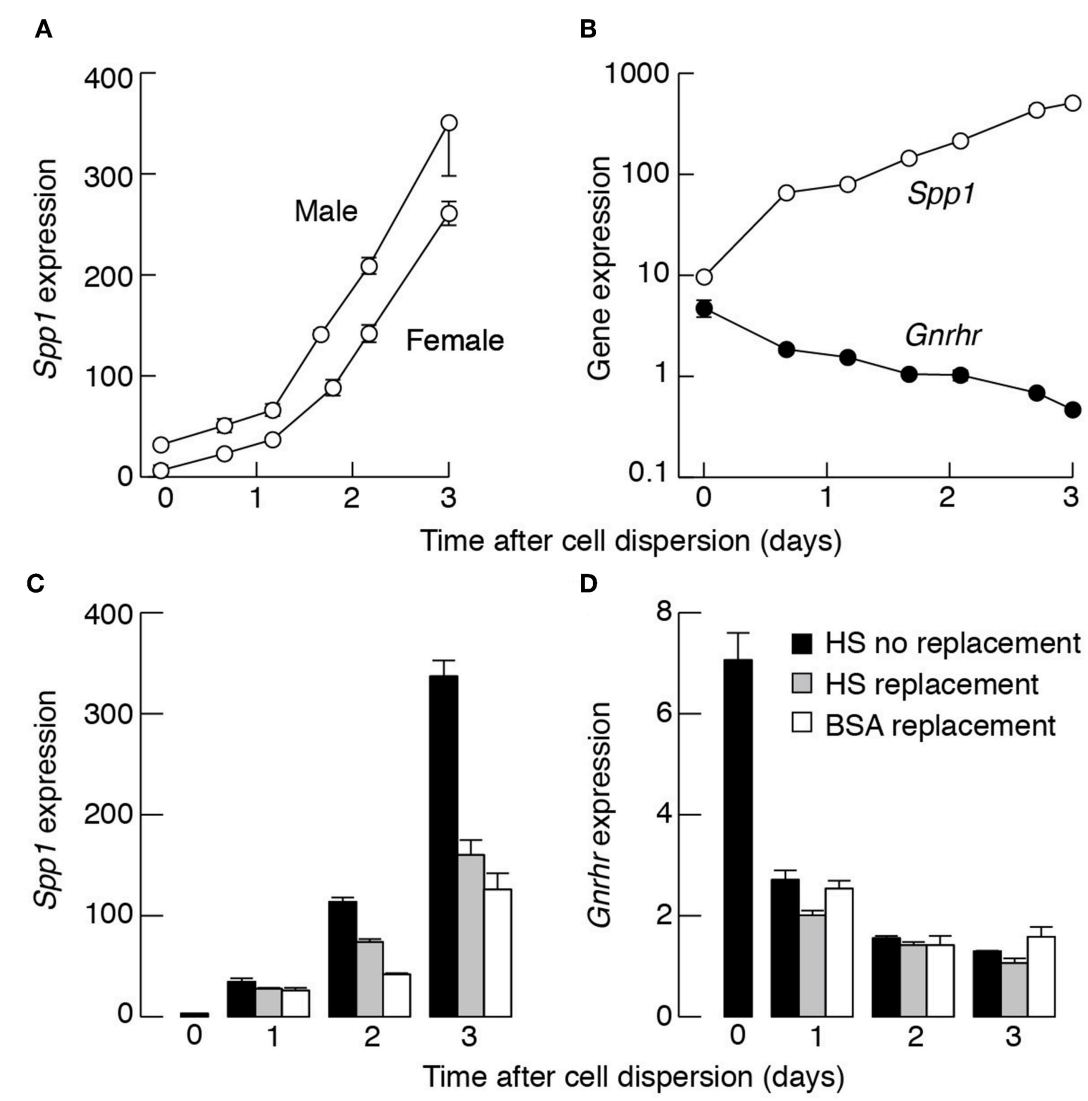
Culturing of dispersed pituitary cells stimulates *Spp1* expression. **(A)** The time course of basal *Spp1* expression in cultured pituitary cells. Cells were derived from 7-week-old female and male rats. Zero indicates *Spp1* expression levels immediately after cell dispersion. Cells were cultured in medium 199 containing horse serum. **(B)** Opposite effects of dispersion and culturing of pituitary cells from females on *Spp1* and *Gnrhr* expression. In **(A)** and **(B)**, cells were continuously cultured in medium 199 containing horse serum without replacement of old medium with fresh. **(C,D)** Effects of replacement of culturing medium on rate of basal *Spp1* **(C)** and *Gnrhr* **(D)** expression. Old media were replaced with fresh media after 12, 36, and 60 h incubation and cells were collected for mRNA extraction immediately after dispersion, and 24, 48, and 72 h after dispersion.

Next, we investigated the expression of basal *Spp1* in primary pituitary cell cultures. After cell dispersion, the *Spp1* expression progressively increased as a function of time in both female and male pituitary cell cultures. The upregulation of *Spp1* expression persisted over several days; Figure 7A illustrates the time course of upregulation of *Spp1* expression during the first 3 days of culturing in medium 199 containing 10% horse serum. Similar growth profiles in *Spp1* expression were observed in cells cultured in medium 199 containing fetal calf serum (data not shown). In cells cultured in serum-free and 0.1% BSA-containing medium, the growth in gene expression was not abolished and the rate of expression was only slightly attenuated. For example, in cells from 7-week old females cultured overnight in horse-serum containing medium or 0.1% BSA-containing medium, the expression of *Spp1* was 53.21 ± 2.52 and 43.88 ± 3.26, respectively. In contrast to *Spp1*, the expression of *Gnrhr* decreased progressively with culturing time [Figure 7B and (38)]. This indicates that loss of pulsatile GnRH stimulation and tridimensional pituitary structure has opposite effect on expression of *Spp1* and gonadotroph marker genes.

The *Spp1* expression in pituitary cells cultured in poly-D-lysine coated wells for 48 h was 112 ± 24, while in collagen-coated wells was 134 ± 36, both relative to *Gapdh* expression (*n* = 6). However, the rate in *Spp1* expression decreased in female pituitary cells cultured in horse serum- and BSA-containing medium when media were replaced with fresh medium once or twice during 72 h incubation (Figure 7C). In contrast, the decay in *Gnrhr* expression was not affected by washing procedure (Figure 7D). These observations are consistent with a hypothesis that an autocrine or paracrine factor, other than GnRH (39), stimulates *Spp1* expression.

### *Spp1* mRNA Expression Is Not Regulated by GnRH

**Figure 8:**
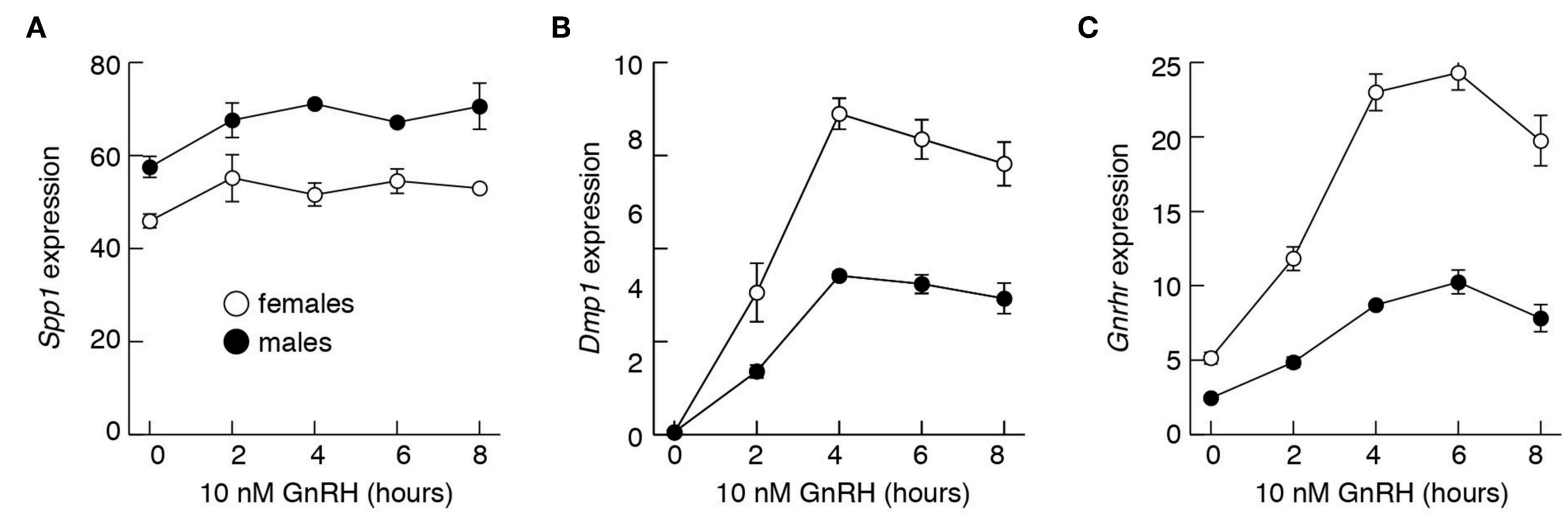
The lack of an ability of GnRH to stimulate *Spp1* expression in cultured anterior pituitary cells. Experiments were performed with cells from 4-week-old females and males. Cells were cultured overnight in medium 199 containing horse serum, which was replaced with BSA-containing medium 199 supplemented with 10 nM GnRH in the morning, cultured for 0 to 8 h, and mRNA was extracted for measurement of *Spp1* **(A)**, *Dmp1* **(B)**, and *Gnrhr* **(C)** expression.

To evaluate effects of GnRH on *Spp1* mRNA expression more directly, we performed two types of experiments, *in vitro* and *in vivo*. Figure 8 summarizes experiments done with pituitary cells derived from 4-week old females and males. Treatment of 20 h-old cultures of these cells with 10 nM GnRH during 8 h incubation did not affect *Spp1* expression (Figure 8A). In contrast, the expression of a sister gene *Dmp1* increased in a time-dependent manner, with a peak in response after 4 h of incubation (Figure 8B). The expression of *Gnrhr* was also stimulated by GnRH in both pituitary cultures, from females and males (Figure 8C).

**Figure 9:**
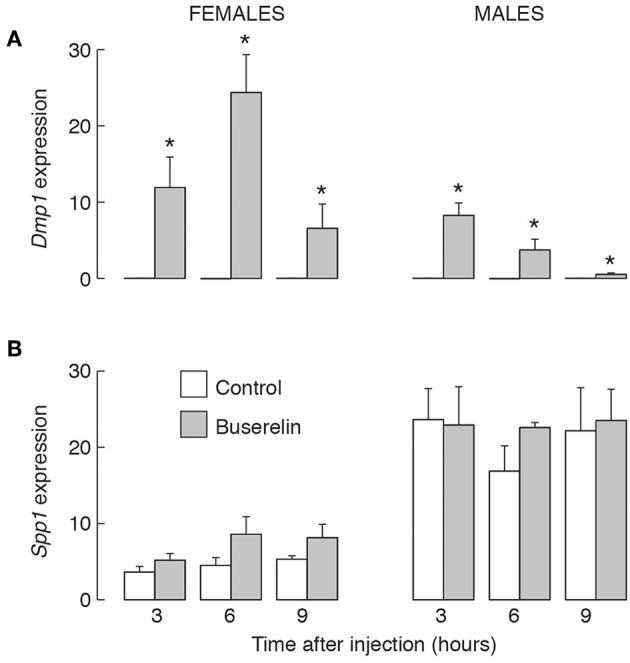
The lack of an effect of GnRHR agonist injection *in vivo* on *Spp1* mRNA expression in anterior pituitary gland. Prepubertal (4-week-old) female and male rats were injected with buserelin acetate, a GnRHR agonist (5 μg/0.4 ml per animal or solvent (0.4 ml PBS) intraperitoneally. Three, 6 or 9 h after injection, anterior pituitary glands were removed and qRT-PCR analysis for *Dmp1* **(A)**, *Spp1* **(B)**, and *Gapdh* was performed; asterisks indicate significant differences vs. the corresponding solvent injected animals; *p* < 0.05 or higher.

The results of *in vivo* experiments with 4-week-old female and male rats were summarized in Figure 9. Animals were intraperitoneally injected with saline solution (solvent) or 5 μg of buserelin acetate, a GnRHR agonist. Animals were sacrificed 3, 6, and 9 h after injection, blood was collected for serum LH measurements and pituitary glands were removed for qRT-PCR analysis. LH measurements confirmed that stimulus secretion coupling was operative under these experimental conditions (data not shown). We also observed a progressive *Dmp1* expression of comparable levels to those observed in cultured pituitary cells (Figure 9A). However, buserelin acetate treatment did not affect *Spp1* expression (Figure 9B).

**Figure 10:**
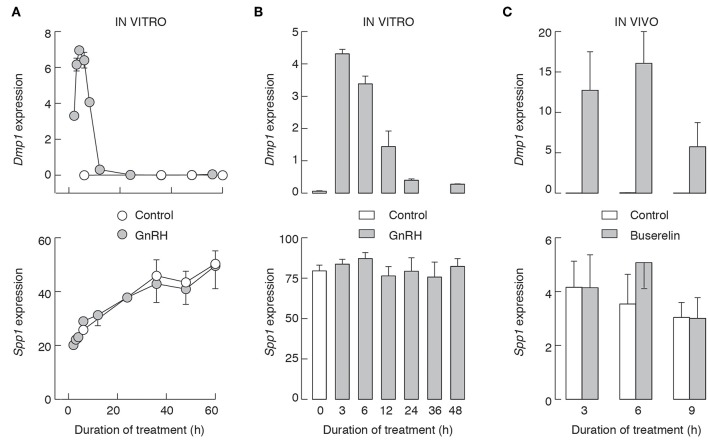
The lack of an effect of GnRHR activation on *Spp1* expression *in vitro* **(A,B)** and *in vivo* **(C)**. Experiments were performed with 7-week-old females. **(A)** Pituitary cells were cultured overnight in medium 199 containing horse serum, washed in the morning, and cultured in medium 199 supplemented with BSA with or without 10 nM GnRH for 0–60 h prior to the end of experiments, and samples were analyzed for *Dmp1* (top) and *Spp1* (bottom) mRNA expression. **(B)** 96 h old cell cultures were treated as described in **(A)**, up to 48 h, and samples were analyzed for *Dmp1* (top) and *Spp1* (bottom) mRNA expression. **(C)** Animals were injected with buserelin acetate or solvent as described above. Gray bars and circles, GnRH/buserelin acetate-treated cells/animals; white bars and circles, solvent-treated cells/animals.

We also performed two *in vitro* experiments using pituitary cells from 7-week-old female rats. In the first experiment, cells were cultured overnight in GnRH-free medium, and after that for 2–60 h in the presence and absence of 10 nM GnRH. Under these conditions, GnRH-induced expression of *Dmp1* (Figure 10A, top), with kinetics comparable to that we reported earlier, with a peak in response observed after 6 h GnRH application, followed by a decay to levels that were on the edge of detection by qRT-PCR (32). The subsequent application of GnRH was inefficient, indicating that decay in *Dmp1* expression is not due to degradation of GnRH but reflects desensitization of response (data not shown). In contrast, during 60 h of incubation, there was a progressive increase in *Spp1* expression in both controls and 10 nM GnRH-treated cells, but no significant differences between treated and untreated cells at the same time points (Figure 10A, bottom).

In the second experiment, pituitary cells were initially cultured for 96 h without GnRH, followed by washing and addition of fresh medium supplemented with 10 nM GnRH. Under these conditions, we also observed a time-dependent induction of *Dmp1* expression by GnRH (Figure 10B, top), indicating that GnRH-induced intracellular signaling and stimulus transcriptional coupling were still operative. However, the expression of *Spp1* was comparable in all groups (Figure 10B, bottom). *In vivo* injected buserelin acetate to 7-week old females also stimulated *Dmp1* expression and did not affect *Spp1* expression (Figure 10C).

*In vitro* experiments were also done with pituitary cultures from 2-, 3-, 6-, 8-, 9-, and 12-week old animals and no change in *Spp1* expression was observed in GnRH (10 nM)-treated cells (data not shown), further supporting the view that GnRH does not regulate *Spp1* expression. We also treated cultured cells with 100 nM thyrotropin-releasing hormone, 100 nM corticotropin-releasing hormone, 100 nM somatostatin-28, 1 μM dopamine, 1 μM oxytocin, 1 μM PACAP28, 1 μM endothelin-1, 25 ng/ml activin, 100 ng/ml IGF, 10 ng/ml EGF, and 2 ng//ml TGFβ1 for 6 h. None of these treatments affected *Spp1* expression *in vitro* (data not shown).

## Discussion

The expression of OPN mRNA in pituitary gonadotrophs has been reported previously (33). Here we provide further evidence that *Spp1* is expressed in gonadotrophs but not in other pituitary cell types. First, the developmental pattern of *Spp1*/OPN is sex-specific and comparable to that observed for *Lhb, Fshb*, and *Gnrhr* (35), the well-established marker genes for gonadotrophs (40). Second, there was no correlation between *Spp1* vs. *Gh1, Prl, Tshb*, and *Pomc*, the marker genes for somatotrophs, lactotrophs, thyrotrophs, and corticotrophs, respectively. Third, immuno-histochemical analysis showed the expression of OPN only in LHβ- and LHβ/FSHβ- positive cells. Our recent single cell RNA-sequence also showed the expression of *Spp1* in gonadotrophs only (NCBI Sequence Read Archive accession SRP151788). Thus, *Spp1* is an additional gonadotroph-specific gene.

The expression of gonadotroph-marker genes, such as *Lhb, Fshb*, and *Gnrhr*, is regulated by GnRH (41). Previously we also showed that pulsatile GnRH application facilitated expression of numerous genes, including *Fshb, Cga*, and *Gnrhr*, but not *Spp1* (32). Here we show that both *in vitro* and *in vivo* activations of GnRHR were ineffective in induction of *Spp1*. We also show that *Spp1* expression varies during estrous cycle, with the pattern not comparable to endogenous GnRH release. Activation of other G protein-coupled receptors expressed in gonadotrophs was also ineffective. In other tissues, the expression of OPN is up-regulated by numerous growth and differentiation factors, including transforming growth factor-β superfamily, bone morphogenic proteins, epidermal growth factors, platelet-derived growth factor, and inflammatory cytokines. Also, steroids, retinoic acid, glucocorticosteroids, and 1.25-dihydroxyvitamin D3 increase OPN expression (42). In our experiments, IGF, EGF, TGFβ1, and activin were ineffective.

We established previously that *Dmp1* is a gonadotroph-specific gene within cells of anterior pituitary gland (32). However, the expression of these two SIBLING genes in gonadotrophs varies. Both genes are expressed *in vivo* in a sex-specific manner, but the expression of *Spp1* was always higher in males and *Dmp1* was always better expressed in females. In postpubertal females, the expression of *Spp1* was largest during the diestrus stage of estrous cycle, whereas the expression of *Dmp1* was most prominent during the late proestrus. In further contrast to *Spp1*, the expression of *Dmp1 in vivo* and *in vitro* was regulated by GnRHR; continuous GnRH application caused a transient stimulation of *Dmp1* expression followed by prolonged desensitization. Thus, although *Dmp1* is a sister gene of *Spp1*, it follows the expression pattern of *Lhb, Fshb*, and *Gnrhr* (32, 41).

The role of pituitary gonadotrophs in reproduction is well-established. Work with expression of SIBLINGs in pituitary gland indicates that gonadotrophs may have an additional cell-type specific function in anterior pituitary gland. At the present time, this function is unknown. Based on functions of OPN and DMP1 in other tissues, we may speculate that these proteins contribute to the proper organization of the cell-ECM tridimensional network, the former in GnRHR-dependent manner and the latter in a GnRHR-independent manner. The work on development of pituitary gland has indicated that, at birth, the pituitary cell types are roughly organized into layers with gonadotrophs being the most ventral (43). However, by adulthood spatial organization of the cell types appears more random (44). It has also been proposed that layering of pituitary cell types at birth could be required to establish networks of specific cell types, rather than a relationship with the timing of cell cycle exit (45). Thus, it is reasonable to speculate the potential role of these proteins in postnatal organization of pituitary.

We also present evidence, for the first time, that *Spp1* expression increased progressively after pituitary cell dispersion in both female and male cultures, reaching 30–40-fold increase in mRNA levels within 3 days. Such response suggests that OPN signals to other pituitary cell types for changes in cell-matrix network structure. *Spp1* is also upregulated as early as 6 h after skin wounding and healing was altered in mice lacking a functional *Spp1*. This and some additional analyses led the authors to conclude that OPN has a role in tissue remodeling and during matrix reorganization after injury (46). In general, OPN has been shown to promote attachment and spreading of a variety of cell types through its glycine-arginine-glycine-aspartate-serine cell binding domain, i.e., OPN can be classified as an adhesive protein (47). Thus, OPN may represent an initial signal for reconstruction of tridimensional structure of pituitary gland.

Consistent with this hypothesis, it has been shown that rat anterior pituitary cells *in vitro* can partly reconstruct the topographic nature of the pituitary gland, which includes few junctional complexes between hormone-producing cells (48). More recently, the same group reported about reassembly of anterior pituitary organization by hanging drop cell culture. Specifically, the authors reported that the topographic affinities of hormone-producing cells were maintained, that folliculostellate cells were interconnected with typical cytoplasmic protrusions to form tridimensional network, with the major ECM components, collagens and laminin, being deposited and distributed around the cells (49). It has also been reported that gonadotrophs can signal to the lactotrophs through the release of a paracrine humoral factor distinct from LH and FSH and in a GnRH-independent manner (50). Further studies indicated that a common alpha subunit of pituitary gonadotropins accounts for influence of gonadotrophs on lactotroph functions (51–53). We may speculate that OPN is another protein released by gonadotrophs, which contributes to the crosstalk among anterior pituitary cells.

Dispersion of pituitary cells could be considered as the stress situation for pituitary tissue. In general, OPN plays a role in immune regulation and stress responses (54). It has also a role in mediating oxidative stress (55), mechanical stress (56), and cellular stress (57). OPN also plays a significant role in the regulation of the hypothalamus-pituitary-adrenal axis hormones in animals exposed to chronic restraint stress (58). Cancer also reflects the loss of tissue organization and aberrant behavior of the cellular components and tumors have been likened to wounds that fail to heal (4). Not surprising, elevated OPN expression has been detected in numerous tumors (59–61). Elevated OPN levels were also detected in silent corticotroph adenomas and non-functioning gonadotroph adenomas (62). OPN expression is inhibitable on the levels of gene transcription and the RNA message, and its protein ligand activity can be blocked with antibodies or synthetic peptides, which led to idea to consider OPN as a candidate target for cancer therapy (63).

For understanding the signaling function of OPN in intact pituitary gland, dispersed pituitary cells, and pituitary tumors, it is critical to identify OPN receptors and cell types expressing these receptors. In general, OPN binds to several integrins, including αv(β1, β3, or β5), and (α4, α5, α8, or α9)β1, and is a ligand for CD44 receptor splice variants, specifically v6 and/or v7 possibly in conjunction with a β1 integrin (63). It is also known that exogenous addition of OPN to OPN-/- osteoclasts increased the surface expression of CD44 (64). The expression of αvβ3 integrin was reported in immortalized GH_3_ lacto-somatotrophs (65). It has also been reported that cultured rat anterior pituitary cells expressed the β1 integrin subunit (11) as well as that integrin β1 signaling is required for the proliferation of folliculostellate cells in rat anterior pituitary gland under the influence of ECM (66). To our best knowledge, at the present time no data exist describing the expression of *Cd44* and its protein in normal mammalian anterior pituitary cells.

In summary, here we provide further evidence for the expression of *Spp1* and *Dmp1* in pituitary gonadotrophs, but not other pituitary cell types, in an age-, sex-, and estrous cycle stage-specific manner. Two genes also differ in regulation of their expression; *Dmp1* expression is regulated by GnRH, whereas *Spp1* expression increases progressively in culturing pituitary cells in a GnRH-independent manner, presumably in response to an unidentified paracrine factor. Further work should be focused on secretion of these two proteins by gonadotrophs under different experimental paradigms, characterization of integrin and CD44 receptors within the secretory and non-secretory anterior pituitary cells and their signaling pathways, and function in pituitary gland.

## Ethics Statement

All experiments were approved by the NICHD Animal Care and Use Committee.

## Author Contributions

SS and IB: conceptualization. IB, MJ, RP, DA, and MK: experimental work. SS: writing—original draft. SS, IB, MJ, and RP: writing—review and editing. SS and IB: data analysis and figure preparation, supervision, and funding acquisition.

### Conflict of Interest Statement

The authors declare that the research was conducted in the absence of any commercial or financial relationships that could be construed as a potential conflict of interest.

## Abbreviations

DMP1: dentin matrix protein 1
ECM: extracellular matrix
FSH: follicle-stimulating hormone
GnRH: gonadotropin-releasing hormone
GnRHR: GnRH receptor
LH: luteinizing hormone
OPN: osteopontin
SIBLINGs: small integrin-binding ligand, N-linked glycophosphoproteins
*Spp1*: secreted phosphoprotein 1.
